# Genetic modifiers of repeat expansion disorders

**DOI:** 10.1042/ETLS20230015

**Published:** 2023-10-20

**Authors:** Sangeerthana Rajagopal, Jasmine Donaldson, Michael Flower, Davina J. Hensman Moss, Sarah J. Tabrizi

**Affiliations:** 1 UCL Huntington's Disease Centre, Department of Neurodegenerative Disease, UCL Queen Square Institute of Neurology, Queen Square, London WC1N 3BG, U.K.; 2 UK Dementia Research Institute, University College London, London WCC1N 3BG, U.K.; 3 St George's University of London, London SW17 0RE, U.K.

**Keywords:** cag repeat, DNA synthesis and repair, genetic modifier, repeat expansion, somatic DNA expansion, somatic instability

## Abstract

Repeat expansion disorders (REDs) are monogenic diseases caused by a sequence of repetitive DNA expanding above a pathogenic threshold. A common feature of the REDs is a strong genotype–phenotype correlation in which a major determinant of age at onset (AAO) and disease progression is the length of the inherited repeat tract. Over a disease-gene carrier's life, the length of the repeat can expand in somatic cells, through the process of somatic expansion which is hypothesised to drive disease progression. Despite being monogenic, individual REDs are phenotypically variable, and exploring what genetic modifying factors drive this phenotypic variability has illuminated key pathogenic mechanisms that are common to this group of diseases. Disease phenotypes are affected by the cognate gene in which the expansion is found, the location of the repeat sequence in coding or non-coding regions and by the presence of repeat sequence interruptions. Human genetic data, mouse models and *in vitro* models have implicated the disease-modifying effect of DNA repair pathways via the mechanisms of somatic mutation of the repeat tract. As such, developing an understanding of these pathways in the context of expanded repeats could lead to future disease-modifying therapies for REDs.

## Introduction

Repeat expansion disorders (REDs) are monogenic diseases caused by an expanded sequence of repetitive DNA. Short tandem repeats (STRs) are a normal constituent of the human genome. They are repeated units of 1–6 base pairs, also termed microsatellites, and make up 3% of our genetic material [1]. STRs are important for centromere and telomere function and are found in intergenic regions, exons, introns and promoters [2]. Only certain STRs are linked with disease [3]. The length of STRs are variable in human populations and STRs expanded over a pathogenic threshold lead to over 50 REDs causing predominantly neurological and developmental disorders. REDs include the polyglutamine diseases which are caused by expanded cytosine–adenine–guanine (CAG) repeats encoding glutamine e.g. Huntington's disease (HD), spinocerebellar ataxia (SCA) 1, 2, 3, 6, 7, and 17, spinal-bulbar muscular atrophy (SBMA), those with other exonic repeats, and many with non-coding expansions e.g. Fragile X disease (FXD) and Myotonic Dystrophy (DM1 & 2) (Table 1) [5]. REDs can arise from families without a history of disease if a germline repeat expansion occurs, leading the repeat to enter the pathogenic range in offspring [4]. Despite being monogenic, individual REDs are phenotypically variable, and it is through exploring what genetic modifying factors drive this phenotypic variability that has illuminated key pathogenic mechanisms that are common to this group of diseases. REDs often have a long pre-symptomatic phase and human studies have shown that the pathogenic process may be influenced during this time by genetic modifiers and environmental factors [5].

**Table 1:** Examples of repeat expansion disorders in coding and non-coding regions

Disease	Gene	Repeat motif	Non-disease repeat	Pathogenic repeat	Location
Huntington disease	*HTT*	CAG	6–35	36–180	Exon 1
Huntington disease like 2	*JPH3*	CAG	<50	>50	Exon 2A
Spinocerebellar ataxia 1	*ATXN1*	CAG	6–35	>39	Exon 8
Spinocerebellar ataxia 2	*ATXN2*	CAG	14–31	37–270	Exon 1
Spinocerebellar ataxia 3	*ATXN3*	CAG	12–44	∼60–87	Exon 10
Spinocerebellar ataxia 6	*CACNA1A*	CAG	≤18	19–33	Exon 47
Spinocerebellar ataxia 7	*ATXN7*	CAG	7–27	37–460	Exon 1
Spinocerebellar ataxia 17	*TBP*	CAG	25–40	49–66	Exon 3
Oculopharyngeal muscular dystrophy	*PABPN1*	GCG	≤10 [16]	11–18	Exon 1
Dentatorubral-pallidoluysian atrophy	*ATN1*	CAG	6–35	48–93	Exon 5
Spinobulbar muscular atrophy	*AR*	CAG	<34	38–68	Exon 1
Spinocerebellar ataxia 10	*ATXN10*	ATTCT	10–32	280–4500	Intron 9
Spinocerebellar ataxia 31	*BEAN1*	TGGAA	8–20 [17]	500–760	Intron/intergenic region
Spinocerebellar ataxia 36	*NOP56*	GGCCTG	5–14 [18]	650–2500	Intron 1
Myotonic dystrophy type 2	*CNBP*	CCTG	<30 [9]	50–11 000	Intron 1
Familial adult myoclonic epilepsy 1	*SAMD12*	TTTCA	<100 [19]	105–3680	Intron 4
Familial adult myoclonic epilepsy 2	*STARD7*	ATTTC	Undetermined [19]	150–460	Intron 1
Familial adult myoclonic epilepsy 3	*MARCHF6*	TTTCA	9–20 [19]	700–1035	Intron 1
Friedreich's ataxia	*FXN*	GAA	<40	Pathological threshold >70 but 600–900 common	Intron 1
Chromosome 9 open reading frame 72 (C9orf72)	*C9orf72*	GGGCC	<20 [20]	Pathological threshold >30 but >300 common	Intron 1
Cerebellar ataxia, neuropathy and vestibular areflexia syndrome (CANVAS)	*RFC1*	AAGGG	11 repeats (allele frequency (AF) = 0.75), 12–200 (AF = 0.13), 40–1000 (AF = 0.08) [21]	400–2000 [22]	Intron 2
X-linked dystonia Parkinsonsim	*TAF1*	AGAGGG (retrotransposon insertion)	Repeat not usually present	>35	Intron 32 [23]
Myotonic dystrophy1(DM 1)	*DMPK*	CTG	5–37	50–10 000	3′UTR
Spinocerebellar ataxia 8	*ATXN8*	CAG	15–50 [24]	74–1300	3′UTR
Fragile X syndrome	*FMR1*	CCG	<45	>200	5′UTR
Neuronal intranuclear inclusion disease	*NOTCH2NLC*	CGG	4–41 [25]	66–517	5′UTR

Pathogenic expansions in coding regions are in general shorter than those in non-coding regions. This list is not exhaustive but is rather to illustrate that larger pathogenic repeat ranges are seen in conditions where the mutation is non-coding compared with those located in coding regions.

Adapted from Chintalaphani et al. [26] and Bunting et al. [27].

There are various mechanisms through which the expanded repeat drives pathology, for example small changes in length can alter gene expression through changing methylation and splicing patterns as well as binding of transcription factors [6, 7]. Translated proteins from expanded STRs often have the propensity to aggregate leading to cellular dysfunction. A common feature of the REDs is a strong genotype–phenotype correlation: longer repeats are correlated with earlier age at onset (AAO) and rate of progression. During a pathogenic gene carrier's lifetime, the inherited repeat tract lengthens in somatic cells in an age-dependent and tissue-specific manner. For example in HD, repeat expansion in post-mitotic tissue, such as striatum, is associated with earlier AAO and increases symptom severity [8–11]. Somatic expansion appears to be a pathogenic rate driver, driving increased toxicity of the resulting cognate protein or RNA [6]. Expansion in germ cells sometimes leads to anticipation, the phenomenon of increasing disease severity and earlier AAO in successive generations. However, there is great variation in how much the AAO can be attributed to repeat length [12] (See Table 2), and a study found that disease duration is independent of repeat length [13]. Studies have identified residual heritability in disease onset and progression after accounting for repeat length. There are genetic modifiers both at the site of the repeat (cis modifiers) and across the genome (trans modifiers). In multiple HD, FXD and DM1 model systems DNA damage repair (DDR) proteins have been shown to drive somatic expansion and recent genome-wide association (GWAS) studies have converged on DDR loci as modifiers of clinical outcomes in many REDs [14, 15]. This review discusses the identification of genetic modifiers of REDs, particularly those affecting somatic expansion, the mechanisms through which they operate and how their study may lead to novel therapies for these devastating and currently untreatable conditions. There are other modifiers that are not yet understood mechanistically or there is insufficient evidence of their significance however this review will not discuss these.

**Table 2:** Genes implicated as causing an altered disease course in repeat expansion disorders

Disorder	Genetic modifier	Effect	Method used to identify genetic modifier
HD	FAN 1	Variants that delay and hasten AAO Rare variant hastens AAO Increased expression associated with delayed AAO and stabilises repeats in *HTT* cell line	GWAS [15] and candidate gene study [12] Candidate exome sequencing analysis [39] Transcriptome-wide association study and *in vitro* model [60]
	MSH3	Variant slows disease progression Increased expression associated with faster progression Increases somatic CAG expansion in striatum	GWAS [55] Transcriptome-wide association study [58] HD homologue mouse model [61]
	MLH1	Variant affecting HD progression	GWAS and candidate genotyping [62]
	PMS1, PMS2, LIG1	Variants altered AAO	GWAS [15]
	MLH3	Variants that delay and hasten disease	Candidate gene study [38]
	MSH6	Reduces intergenerational contractions	HD homologue mouse model [61]
	MSH2	Deficiency tended towards contractions	Mouse HD model [63]
DM1	MSH3	Variant reduced somatic expansion Variant associated with increased somatic instability Deficiency of MSH3 reduced somatic instability Deficiency of MSH3 reduced intergenerational expansion	Transcriptome-wide association study [58] Genotyping candidate genes in DM1 cohort [49] Humanised DM1 mouse model [64] Humanised DM1 mouse model [65]
	MSH6	Deficiency increased CTG repeat instability in mice	Humanised DM1 mouse model [64]
	MSH2	Knock out shifted instability towards contractions MSH2 knock out prevent germline expansions	MSH2 deficient mouse [66] MSH2 deficient mouse [67]
SCA 1	FAN1	Variants that delay and hasten AAO	Candidate gene study [12]
	PMS2	Variants that delay and hasten AAO	Candidate gene study [12]
	TBP, ATN1 & *HTT*	Associated with altered AAO	Genotyping of CAG genes in SCA1 cohort [68]
SCA2	FAN1	Variants that delay and hasten AAO	Candidate gene study [12]
	PMS2	Variants that delay and hasten AAO	Candidate gene study [12]
SCA3	MLH1	Variant associated with later AAO but did not reach significance threshold	GWAS [69]
	RAG	Variant associated with hastening AAO	GWAS [69]
	TRIM29	Variant associated with hastening AAO	GWAS [69]
	FAN1	Variants that delay and hasten AAO Variant hastened AAO	Candidate gene study [12] Candidate gene study [57]
	PMS2	Variants that delay and hasten AAO	Candidate gene study [12]
SCA6	FAN1	Variants that delay and hasten AAO	Candidate gene study [12]
	PMS2	Variants that delay and hasten AAO	Candidate gene study [12]
	RRM2B	Variants that delay and hasten AAO	Candidate gene study [12]
SCA7	FAN1	Variants that delay and hasten AAO	Candidate gene study [12]
	PMS2	Variants that delay and hasten AAO	Candidate gene study [12]
SCA17	FAN1	Variants that delay and hasten AAO	Candidate gene study [12]
	PMS2	Variants that delay and hasten AAO	Candidate gene study [12]
Fragile X	FAN1	Knock out shows increased somatic expansion SNP associated with increased somatic expansion	FXS mouse model with FAN1 knock out [70] Candidate SNP analysis [59]
	MLH3	Point mutation in endonuclease domain reduces somatic expansion Required for germline and somatic expansion	FXS embryonic mouse stem cell model [71] FXS mouse model [58, 72]
	EXO1	Mutation results in increased germline and somatic expansions	FXS mouse model [73]
	MSH3	SNP associated with altered levels of somatic expansion	Candidate SNP analysis [59]
	MSH2	Mutation associated with increased somatic instability. Loss of one MSH2 allele reduced intergenerational expansion	FXS mouse with MSH2 mutation [74]
	MutLα, MutLγ & MutLβ	All required for somatic expansion	FXS mouse embryonic stem cell model [75]
	Polβ	Heterozygous mutation in Polβ reduced expansion in paternal gametes and somatic expansion	FXS mouse model [76]
	CSB	Mutation in CSB suggests that transcription coupled repair may have a role in tissue instability	FXS mouse model [77]
FRDA	MSH2 & MSH6	Absent MSH2 & MSH6 in mice reduced somatic instability	FRDA transgenic mouse model [78]
	PMS2	Reduces large expansions	FRDA transgenic mouse model [78]
	MLH1	Loss of MLH1 activity reduced somatic and intergenerational expansion	MLH1 deficient FRDA mouse model [79]
X- linked dystonia Parkinsonism	MSH3, PMS2, DHFR, ANKRD6, EIF2AK1	Variants associated with altered AAO	GWAS [80]

This table shows genetic modifiers identified in many REDs that effect somatic instability. At many loci there are signals with different directions of effect.

## Tandem repeat length

HD shows autosomal dominant inheritance and is caused by a CAG expansion in exon 1 of the *huntingtin* (*HTT)* gene. Like other REDs, inherited CAG repeat length is the main determinant of AAO. Epidemiological studies in HD have established complete penetrance for clinical manifestations of HD with an inherited CAG repeat tract of ≥40 [28]. Those with ≥56 repeats usually develop juvenile HD with a more Parkinsonian phenotype, seizures and rapid progression [29]. 36–39 CAGs shows incomplete penetrance where 38 and 39 repeats confer around 60% and 90% risk of penetrance by the age of 82, respectively [28]. 27–35 CAG repeats are termed intermediate alleles where carriers show no clinical symptoms but are at risk of germline expansion in their offspring [29, 30].

Pathogenic expansions are longer in diseases where expansions are non-coding [31, 32] (Table 1). FXD is an X-linked dominant disorder caused by a CGG non-coding repeat in the 5′UTR of the *FMR1* gene which in health has an unmethylated repeat of up to 45 repeats [33]. Expansions over 200 repeats leads to hypermethylation and silencing of the gene. The FMR protein is important for CNS synaptic plasticity and its loss leads to a syndrome of intellectual disability and autism. However, an allele with a premutation of 55–200 repeats leads to a late onset Fragile X-tremor/ataxia syndrome in men while women may have primary ovarian insufficiency. This premutation allele shows enhanced promotor activity creating toxic RNA products [34]. Therefore, repeat size determines downstream cellular cascades with vastly divergent clinical phenotypes.

Determining the pathogenic threshold is more uncertain in some REDs. C9orf72 is caused by an intronic GGGGCC repeat and can lead to a frontotemporal dementia (FTD) and amyotrophic lateral sclerosis (ALS). It is the most prevalent genetic cause in both disorders. Generally, the disease range for C9orf72 is >30 repeats with normal being <20 [20], though pathogenic repeats are greater than 400 and often in the 1000s in the disease population. Expansions have been found in 0.15% of controls in a UK 1958 birth cohort and >32 repeats have been shown in those with disease and in controls [35] raising the question of penetrance. However, sizing of larger repeat units is difficult using conventional PCR based methods leading to difficulties in establishing length-phenotype relationships.

## Repeat tract structure

In REDs, the repeat length is the primary determinant of AAO but the pathogenic tract purity is also important. Around 3–5% of DM1 families have GGC, CCG and CTC interruptions in the CTG repeat tract which can lead to slower disease progression with a lack of typical clinical features of DM1 [36]. These interruptions are thought to stabilise the expansion and demonstrated to be associated with reduced somatic instability [28, 37].

In HD, the CAG repeat typically ends with a CAACAG cassette (both CAG and CAA encode glutamine). In a cohort of over 700 HD gene carriers, ultra-deep sequencing showed that over 95% of *HTT* alleles had the typical structure [38]. Loss of CAA interruptions are associated with earlier AAO, whilst additional interruptions (CAA/CAC) are associated with later AAO than predicted by their polyglutamine repeat lengths [26, 39, 40]. After correcting for pure CAG length, deletion of the CAACAG cassette was associated with AAO 10 years earlier than the median HD onset for a typical HD allele. When this occurs in those with intermediate alleles (CAG of 36–39), the chance of clinical symptoms is increased. Conversely, most with duplication of the cassette showed slower disease progression scores. Therefore AAO correlates better with length of the uninterrupted CAG sequence than the number of coded glutamines. It was established that these variants in the repeat tract underly the AAO modifying loci on chromosome 4 proximal to the *HTT* locus identified in a genome-wide association study [15]. When uncorrected for pure CAG length, the loss of interruption variant was associated with increased somatic expansion in blood, likely due to altered stability of secondary structures formed by the expanded repeats such as hairpins [40]. Such structures may form when DNA is single stranded e.g. during transcription and occur when complementary bases on the same DNA strand pair within the repeat sequence. These structures are prone to single-strand breaks, predisposing to errors during DNA repair. This may lead to somatic expansion of repeats, discussed below. Emerging data in HD also suggests that the sequence variation around the repeat in the adjacent polyproline tract may also exert phenotypic effects [41, 42].

Repeat tract interruptions are associated with altered AAO in other REDS including FXS, Friedreich ataxia (FRDA) and SCA 1 and 2 [43–45]. 95% of the wild-type *FMR1* alleles have one or two AGG interruptions in the CGG repeat, both non-coding [46]. A study involving 1471 maternal premutation alleles found that 97.5% of those with intergenerational expansion had no AGG interruptions while in those with two or more AGGs, 46.7% showed intergenerational expansion. A similar pattern was found in those with pathogenic expansions [47].

## Somatic instability of repeats

Somatic expansion describes expansion of the repeat tract in somatic tissue [10, 48]. In a study which examined post-mortem brain tissue of 48 HD patients, after accounting for inherited repeat length, greater somatic expansion of CAG repeats was seen in those with earlier onset [9]. This is concordant with HD blood DNA data where higher degrees of somatic instability has been linked directly to earlier AAO and faster disease progression [38]. In DM1, the *DMPK* CTG repeat exhibits somatic expansions in an age-dependent, tissue-specific (e.g. muscle) and expansion-biased manner and is associated with worse clinical outcomes [49, 50]. C9orf72 also displays similar characteristics with somatic expansions in both neuronal and non-neuronal tissues though correlation with clinical phenotypes is not well delineated [9].

In HD, somatic instability occurs in post mitotic neurons particularly in the striatum. Neuronal tissue is susceptible to DNA damage as it is highly metabolically active and high levels of free radicals are generated [42]. HD has a predilection for the striatum which shows particularly high metabolic demand [9, 27]. Interestingly, the tissue-specific patterns of CAG expansion do not always predict pathology. High instability was observed in the cortex and caudate, which demonstrate vulnerability in HD but are relatively unaffected in SCA [27]. Particular cell types may have unique pathogenic thresholds for different toxic species. Recent single-cell RNA sequencing has demonstrated differential vulnerability of CAG expansion of different cortical cell layers in HD [52].

## Human genetic data and repeat expansion disease

While the existence of somatic instability of the repeat tract of REDs had been well established, more recent human genetic studies and genome wide association studies (GWAS) have had a pivotal role in understanding what genetic modifiers may drive this. These, among other studies, have highlighted the importance of mismatch repair (MMR) and FAN1 in somatic instability (Table 2). The Genetic Modifiers of Huntington's Disease Consortium (GeM) conducted a series of GWAS, first examining the residual age of onset of HD after controlling for CAG repeat length [53]. A significant association at a chromosome 15 locus linked to the DNA repair gene FAN1 displayed two independent effects that accelerate or delay onset by 6.1 years and 1.4 years, respectively. The 2019 GeM consortium strengthened previous findings with significant GWAS signals underpinned by at least six identified candidate modifier loci, many of which contained genes involved in DNA maintenance [15]: PMS1, MLH1, MSH3, PMS2, FAN1, LIG1. The identification of such genes strongly point towards the MMR pathway as an important genetic modifier, likely through involvement in somatic expansion. Other signals were found such as at TCERG1, RRM2B, CCDC82, SYT9, GSG1L that may be related to other mechanisms, or more indirectly involved in DNA maintenance. Subsequent detailed analysis of *FAN1* locus modifiers indicates the presence of two onset-hastening missense changes and an onset-delaying variant associated with greater FAN1 expression [54]. Furthermore, an *MSH3* SNP in HD with genome-wide significance in meta-analysis was associated with a slower progression score in 216 subjects [55]. An association with MMR in a pathway analysis suggested shared mechanisms influence AAO and disease progression, supported by the finding that about 2/3 of the rate of functional, motor and cognitive progression in HD is determined by the same factors that also determine AAO [56]. While DNA maintenance modifier mechanisms are common to different sub-phenotypes of HD, individual modifier effects act preferentially in the motor or cognitive domains [14].

These HD studies raised the question whether DNA damage response mechanisms were specific to *HTT*, or was a broader mechanism relating to repeat expansions. A candidate gene study found an association between 22 DNA repair loci SNPs and AAO in 1462 subjects with polyglutamine SCAs and HD (*P* = 1.43 × 10^−5^) and significant associations at individual SNPs [12]. Additionally, significant association between a FAN1 SNP rs3512 and onset in SCA3 was demonstrated [57]. Supporting this, an allele in *MSH3* exon 1 at the site of the genome-wide signal [55] is associated with a relatively lower rate of somatic CAG·CTG expansion in blood and delayed disease age of onset for HD and DM1 [58]. SNPs in both *MSH3* and *FAN1* are also significantly associated with somatic expansion risk in FXS [59].

## DNA repair mechanisms in REDs

Evidence suggests MMR pathways drive somatic expansion of repeats in HD (CAG), DM1 (CTG), FRDA (GAA) and FXS (CGG) (Table 2). MMR driven mutability is however not always pathogenic as it is harnessed for immunoglobulin hypermutations for the production of high affinity antibodies [81]. Deficiency of core MMR factors, such as MSH2, MSH6, MLH1 and PMS2 have been associated with cancer and can result in genome-wide microsatellite instability (MSI). REDs demonstrate locus-specific expansions at the site of a pathogenic tandem repeats [82, 83]. The mechanisms mediating REDs and cancer are different and highlight the double-edged sword of MMR. The generation of cancer may involve dysfunctional MMR proteins such as loss-of-function mutations in MSH2, MSH6, MLH1 and PMS2 which may be sporadic, or inherited such as in Lynch syndrome [84]. Meanwhile in REDs, functional MMR machinery acts in an error-prone manner on pathogenic repeat sequences, resulting in expansion [89]. The DNA damage response is not the only cellular machinery exhibiting altered function due to the presence of pathogenic repeats as these repeats also trigger non-AUG translation [85].

**Figure 1.**
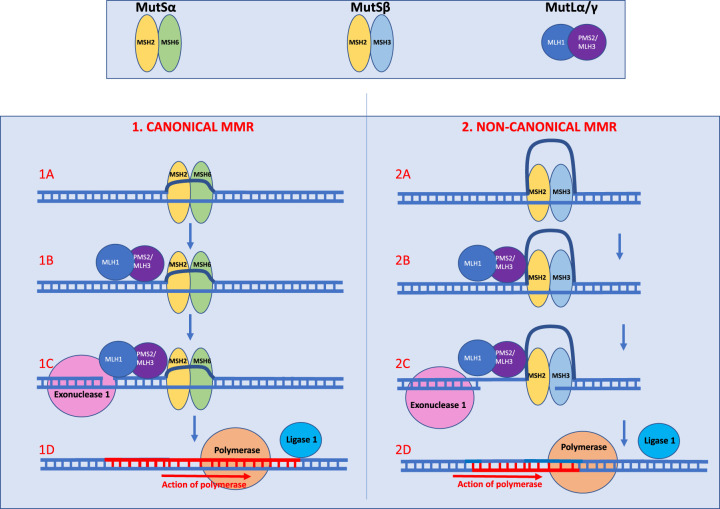
Normal MMR vs mechanisms that may lead to expansion of repeats in non-dividing cells. The figure illustrates the working model based on the current strongest evidence. **The MutS and MutL complexes:** There are two MutS complexes; MutSα, which contains MSH2/MSH6 or MutSβ comprising MSH2/MSH3. MutSα preferentially recognises and targets mismatches and 1–4 base INDELS while MutSβ targets medium-sized loop repair [91]. There are three MutL endonuclease complexes; MLH1 complexed with either PMS2, MLH3 or PMS1 creating MutLα, MutLγ and MutLβ, respectively. MLH3 is involved in meiotic repair but can compensate a small amount for MutLα while PMS1's role is unclear and cannot compensate for MutLα. When DNA damage occurs, in canonical MMR, the MutL complexes’ endonuclease is thought to create a DNA break in the strand with a mismatch (which is assumed to be the strand carrying an incorrect base). MutL induced breaks are the initiation sites for strand excision performed by exonuclease 1. Canonical and Non-Canonical MMR Hypothesis in non-dividing cells (**1**) **Canonical MMR:** (**1A**) The figure illustrates the reaction of MMR to a small loop out. In canonical MMR mismatches, small loops and insertions and deletions (INDELSs) are resolved by the recruitment of one of two MutS complexes; MutSα or MutSβ. MutSα preferentially recognises and targets mismatches and 1–4 base INDELS while MutSβ targets medium loop repair [33, 91]. This occurs when cells are dividing or the DNA is transcriptionally active causing it to unwind and become single stranded. (**1B**) Once bound, the MutS complex induces recruitment of MutL endonuclease complexes. MutLα is the principle MutL complex for most MMR. The MutL complex creates a DNA break in the strand with an existing break. (**1C**) Excision is then performed by exonucleases e.g. exo 1. (**1D**) There is then faithful repair involving DNA polymerase using the opposite strand as the template strand. (**2**) **Hypothesised Non-Canonical MMR:** (**2A**) Strand separation during transcription (or replication) permits pathogenic repeat sequences to form secondary structures e.g. hairpins, R-loops and cruciforms. This figure illustrates the potential downstream effects of large loops. It is thought that these structures act as the substrate for non-canonical MMR. Large loops of 2–10 bases can only be resolved through recognition by MSH3 in MutSβ. (**2B**) The MutL complex is then recruited and unlike in canonical MMR, MutL complex erroneously creates a break in the strand opposite the loop. (**2C**) One hypothesis is that FAN1 nuclease cuts the strand opposite the loop i.e. the complementary strand (though the location of its action has not been fully elucidated). Therefore the strand with the loop is used as the template strand. (**2D**) There is then erroneous resolution of the loop resulting in elongation of the repeat sequence as Polymerase uses the strand with the loop as the template strand, thus incorporating new repeats into the gene.

In dividing cells, MMR operates during DNA replication and is intimately involved with the replication fork as a signal for strand specificity. In the context of DNA damage, the canonical MMR pathway consists of base mismatch recognition, strand specific endonuclease mediated nicking at a site downstream of the mismatch, single-strand degradation and finally single strand synthesis by a polymerase (Figure 1) [86, 87]. Expanded repeat sequences can form secondary structures e.g. hairpins and R-loops and it is thought that these structures act as the substrate for MMR acting erroneously leading to somatic expansion of pathogenic repeats. MutSβ (MSH2–MSH3) likely recognise these structures and recruit MutLα (MLH1–PMS2) or MutLγ (MLH1–MLH3) endonucleases to co-ordinate excision. MutSβ is thought to compete with FAN1 for loop-out structures resulting in expansion or contractions, respectively [88, 89].

Elucidation of MMR in REDs has been aided by genetic knock-outs. Knock-out of both *Msh2* and *Msh3* ablated repeat instability in mouse models of HD [61, 92–94], DM1 [64, 65], FXD [74] and FRDA [78], implicating MutSβ as an essential driver of somatic expansion. Polymorphisms in *Msh3* found between HD mouse strains appeared to mediate somatic expansion rates via differential Msh3 expression [92]. In DM1, a human polymorphism proximal to the ATPase domain of *MSH3* was associated with reduced somatic instability in blood [49] and cellular models of HD revealed the ATPase of MSH3 to be critical in driving repeat expansions [95]. The ATPase catalysis leads to conformational change enabling the sliding of MSH3. Therefore, the MutSβ complex appears to be significant in somatic instability and is known to be important for large loop repair (Figure 1). Interestingly, *MSH3* is known to tolerate loss-of-function variation and therefore represents an attractive therapeutic target [96].

Evidence shows variants in *MLH1* are associated with altered AAO in HD, SCA3 and FRDA. Concordant with hypotheses, MLH1 promotes the expansion of both *HTT* CAG [60, 97, 98] and *FXN* GAA repeats [79, 99]. MutL cofactors *PMS1* and *PMS2,* but not *MLH3* have been implicated by HD GWAS, though experimental data exploring their role in somatic expansion is inconsistent [14, 15]. Variants in *MLH3* are associated with somatic instability in blood of people with HD [38], and is required for *HTT* CAG repeat expansions in *Hdh^Q111^*mice [97, 100] and *FMR1* CCG expansions in *FXD* mice and embryonic stem cells [70, 71, 75].

Although *PMS1* modifies AAO in HD, the molecular functions of PMS1 or the heterodimeric complex it forms, MutLβ (MLH1–PMS1) are not well understood. Only one study has assessed the effect of PMS1 on somatic instability, where PMS1 knockout led to repeat CCG repeat stability in an FXD mouse embryonic stem cell (mESC) model [75]. Loss of PMS2 attenuated repeat instability DM1 mice and FXD mESCs, yet potentiated large GAA *FXN* expansions in an FRDA mouse model [78].

The most significant modifiers of HD AAO are at *FAN1* loci which, although not a canonical MMR factor, it is a structure-specific 5′ exo/endonuclease involved in DNA repair, particularly inter-strand cross-link repair and replication fork recovery [14, 53, 55]. FAN1 likely modifies AAO in HD and SCAs via an effect on somatic instability [12, 53]. Indeed, FAN1 protects against repeat expansions in multiple models of HD [39, 60, 98] and a mouse model of FXD [70]. Knock-out of *Mlh1* and *Fan1* ablated somatic expansion in *Hdh^Q111^* mice suggesting that FAN1 works in an MMR-dependent manner to protect against repeat expansions [98].

FAN1 is hypothesised to stabilise the repeat via two distinct functions. The first function of FAN1 is its interaction with MLH1 through competition with MutSβ for interaction with MutLα [89, 101]. Recent data indicates a protein motif on FAN1, dubbed the SPYF motif, is critical for MLH1 binding and conferring *HTT* CAG repeat stability, likely through its competition with MSH3 [89]. Secondly, via its nuclease activity, it is thought to promote accurate repair at the repeat [39, 102]. Supporting this, exome sequencing revealed rare non-synonymous coding variants clustering to the nuclease domain associated with an earlier AAO [39]. Candidate gene studies in the SCAs and FXD have also implicated a role for FAN1 but further work in model systems is required [12].

These investigations implicate non-canonical DDR directing somatic instability. Of therapeutic interest are strategies to target MSH3 since its knockdown appears not to be particularly oncogenic. Interventions could include MSH3 knockdown using siRNA or antisense oligonucleotides, ATPase inhibition, or potentially involve histone deacetylase 3 (HDAC) inhibition of MSH3 whose activity potentiates somatic expansions in HD mice [103]. Further work is needed to understand how these processes protect against sequence elongation.

## Conclusions

Repetitive DNA sequences are an important source of genetic diversity in healthy human populations [104]. The REDs are a set of monogenic diseases caused by expanded repeat sequences leading to a group of predominantly developmental and neurological abnormalities. The cognate gene in which the pathogenic expansion exists, and whether repeats are intronic or exonic, exerts an effect on pathogenic repeat length threshold and phenotype. Inherited repeat length is a key factor influencing AAO but does not completely explain variability in disease course. GWA studies probing this residual phenotypic variability have identified other genetic modifiers, including an important role for DNA repair, and specifically the mismatch repair pathway. Biochemical, cell and animal models show variants in DNA repair genes underlie repeat instability and influence disease course in numerous REDs, including HD, FXD, SCAs and DM1, suggesting therapeutic intervention could benefit a range of diseases. Recent studies suggest that abnormal DNA structures, such as hairpins, can form at repeat tracts and act as substrates for error-prone non-canonical DDR, resulting in repeat instability. There are not yet any disease-modifying therapies for REDs, but the identification of genetic modifiers demonstrates that disease course can be altered, and give considerable hope that therapeutics harnessing DNA repair, which are currently in clinical development, can delay onset and slow progression in a whole range of REDs. Future therapy design will need to balance the oncogenic risks with RED disease modification but targeting MSH3 is likely to hold some promise.

## Summary

- The REDs are a group of monogenic diseases that lead to a heterogenous group of predominantly developmental, and neurological diseases.
- Repeat lengths in REDs are key to determining the age of symptom onset: larger repeats lead to earlier onset and worse disease severity.
- The purity of repeat tracts impacts on age of onset: the presence of interruptions in repeat sequences leads to less severe disease phenotypes.
- DNA damage repair (DDR) pathways modify disease course by acting through somatic instability of repeat sequences during the lifetime of gene carriers.
- There is a huge therapeutic focus on DDR pathways in REDs which will likely lead to new clinical trials and therapies in the REDs.

## Open Access

Open access for this article was enabled by the participation of University College London in an all-inclusive *Read & Publish* agreement with Portland Press and the Biochemical Society under a transformative agreement with JISC.

## Abbreviations

AAO: age at onset
DDR: DNA damage repair
FRDA: Friedreich ataxia
FXD: Fragile X disease
GWAS: genome-wide association
HD: Huntington's disease
HTT: huntingtin
MMR: mismatch repair
REDs: repeat expansion disorders
SCA: spinocerebellar ataxia
STRs: short tandem repeats
